# Valence-dependent influence of serotonin depletion on model-based choice strategy

**DOI:** 10.1038/mp.2015.46

**Published:** 2015-04-14

**Authors:** Y Worbe, S Palminteri, G Savulich, N D Daw, E Fernandez-Egea, T W Robbins, V Voon

**Affiliations:** 1 Behavioural and Clinical Neuroscience Institute, University of Cambridge, Cambridge, UK; 2 Institute of Cognitive science, University College of London, London, UK; 3 Laboratoire des Neurosciences Cognitives, Ecole Normal Supérieure, Paris, France; 4 Department of Psychiatry, University of Cambridge, Cambridge, UK; 5 Center for Neural Science and Department of Psychology, New York University, NY, USA; 6 Cambridgeshere and Peterborough NHS Foundation Trust, Cambridge, UK; 7 Department of Psychology, University of Cambridge, Cambridge, UK

## Abstract

Human decision-making arises from both reflective and reflexive mechanisms, which underpin goal-directed and habitual behavioural control. Computationally, these two systems of behavioural control have been described by different learning algorithms, model-based and model-free learning, respectively. Here, we investigated the effect of diminished serotonin (5-hydroxytryptamine) neurotransmission using dietary tryptophan depletion (TD) in healthy volunteers on the performance of a two-stage decision-making task, which allows discrimination between model-free and model-based behavioural strategies. A novel version of the task was used, which not only examined choice balance for monetary reward but also for punishment (monetary loss). TD impaired goal-directed (model-based) behaviour in the reward condition, but promoted it under punishment. This effect on appetitive and aversive goal-directed behaviour is likely mediated by alteration of the average reward representation produced by TD, which is consistent with previous studies. Overall, the major implication of this study is that serotonin differentially affects goal-directed learning as a function of affective valence. These findings are relevant for a further understanding of psychiatric disorders associated with breakdown of goal-directed behavioural control such as obsessive-compulsive disorders or addictions.

## Introduction

Flexible behaviour is crucial for adapting to the environment. When choosing an action, we use multiple strategies to obtain potential reward and to avoid potential punishment. Studies on humans and other animals suggest the existence of ‘reflective' or goal-directed responses that depend on prospective consideration of future actions and their consequent outcomes in contrast to ‘reflexive' or habitual responses that relies on retrospective experience with good and bad outcomes.^1, 2, 3^

Computationally, two behavioural control systems have been proposed to arise from different learning algorithms, model-based and model-free learning.^3, 4^ Specifically, a model-based strategy was linked to the goal-directed behavioural control, whereas a model-free strategy, which presumes choices based on previously reinforced actions, suggests shared similarities with habitual control.^3^ Nevertheless, it is likely that habitual behaviour exceeds a simple reinforcement learning model-free mechanism.^5^

These two (often competitive) behavioural control strategies may depend on distinct neuronal systems, and more specifically on limbic (model-free) and on cognitive (model-based) corticostriatal circuits.^1, 6, 7^ Chemical neuromodulation of these systems by the ascending monoaminergic projections has only recently been addressed. Namely, numerous studies have focused on the role of dopamine (DA) as a signal of positive prediction error in model-free learning.^8, 9, 10^ Interestingly, administration of the dopaminergic precursor, levodopa, to healthy volunteers shifted behavioural performance to a model-based over a model-free strategy.^11^

In contrast, the question whether serotonin (5-hydroxytryptamine, 5-HT), another monoamine neurotransmitter, influences the degree to which behaviour is governed by either model-based or model-free systems has not been previously addressed. Serotonin is sometimes considered to be in an opponent, or alternatively in a synergistic, functional relationship with brain DA with respect to behavioural choice.^12^

Recent data show that manipulation of 5-HT can selectively produce effects on both appetitive and aversively motivated behaviour.^13, 14^ Consequently, 5-HT might influence the degree to which behaviour is governed by either model-based or model-free systems in both reward and punishment conditions.

In particular, selective activation of 5-HT neurons of the raphé nucleus promoted long-term optimal behaviour by facilitating waiting for the delayed rewards.^15, 16^ In contrast, low serotonin increased delayed reward discounting.^17^ Consequently, lower serotonin neurotransmission may affect the prospective consideration of behavioural choices and consequently shift the balance between two behavioural controllers towards model-free behaviour. Under punishment, lowering of serotonin levels promoted lose-shift associative learning^18, 19^ and reduced the pavlovian inhibitory bias to aversive stimuli,^20, 21^ which potentially might shift the balance towards goal-directed behaviour.

**Figure 1:**
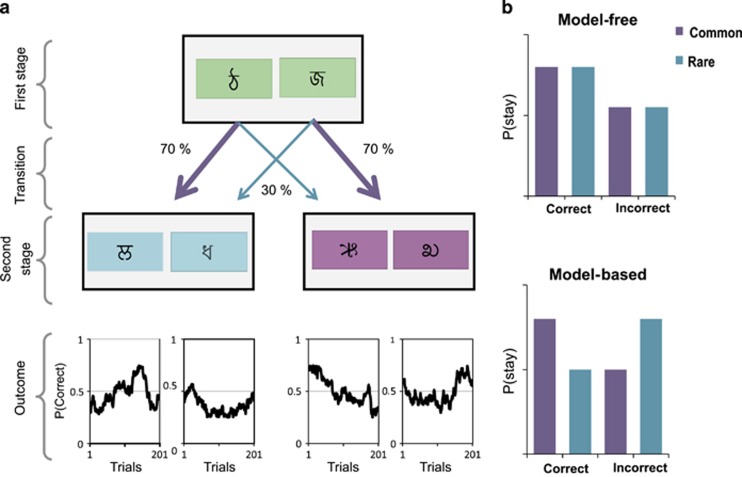
Two-stage decision-making task. Task. (**a**) On each trial (first stage), the initial choice between two stimuli (left-right randomised) led with fixed probabilities (transition) to one of two pairs of stimuli in stage 2. Each of the four second-stage stimuli was associated with probabilistic outcome: monetary reward in the reward or loss in the punishment version of the task. All stimuli in second stage were associated with probabilistic outcome, which changed slowly and independently across the trials. (**b**) Model-based and model-free strategies predict different choice patterns by which outcome obtained after the second stage affected subsequent first-stage choices. In the model-free system, the choices are driven by the reward or the no loss, which increase the chance of choosing the same stimulus on the next trial independently of the type of transition (upper row). In a model-based system, the choices of the stimuli on the next trial integrate the transition type (lower row).

To test these hypotheses formally, we designed a novel version of a model-based versus model-free paradigm based on a two-step sequential choice task^22^ that dissociated the reward and punishment conditions. This task discriminates model-based and model-free behavioural strategies (Figure 1a). On each trial in stage 1, subjects made an initial choice between two stimuli, which led with fixed probabilities to one of two pairs of stimuli in stage 2. Each of the four second-stage stimuli was associated with probabilistic monetary reward (in the reward version of the task) or loss (in the punishment version of the task) (Figure 1a and Supplementary Experimental Procedures). As shown in Figure 1b, model-based or model-free learning are theoretically predicted to produce different patterns by which the events on a trial affect the subsequent first-stage choice. In particular, considering the first-stage choice (stay or shift) as a function of two factors, the transition probability (common or rare) and outcome (reward or punishment), model-free reinforcement learning predicts only a main effect of outcome, whereas the signature of model-based reinforcement learning is an interaction of reward by transition probability. Previous studies on healthy volunteers have shown an intermediate pattern (i.e., using both model-based and model-free strategies) of choice preference on this task, supporting evidence for both behavioural strategies.^22^

To influence serotonin neurotransmission, we used the dietary acute TD procedure in healthy volunteers, which induces a selective and transient reduction of central 5-HT in the human brain.^23, 24, 25^

## Methods

### Experimental procedure

#### Session

A total of 44 participants were assigned to receive either the TD or the placebo (BAL) mixture in a randomised, placebo-controlled, double-blind order (Supplementary Information 1). They were asked to abstain from food and alcohol 12 h before the testing session. Upon arrival, participants completed questionnaires, gave a blood sample for the biochemical measures and ingested either the BAL or the TD drink. To ensure stable and low tryptophan (TRP) levels, behavioural testing was performed and the second blood sample was taken after a resting period of 5 h.

### TD and biochemical procedures

TRP was depleted by ingestion of a liquid amino acid load that did not contain TRP but did include other large neutral amino acids (LNAAs) (see Supplementary Information 2 for biochemical composition of mixtures). Plasma total amino acid concentrations were measured by means of high-performance liquid chromatography with fluorescence end-point detection and precolumn sample derivatisation. The TRP:ΣLNAAs ratio was calculated as an indicator of central serotoninergic function.^25^ The obtained values were entered in repeated measures analysis of variance (ANOVA) with time as a dependent factor and group as an independent factor.

### Task

We used the two-stage decisional task with separate reward and punishment conditions (Supplementary Information 3). The reward version of the task was identical to the previously published task by Daw *et al.*^22^ Briefly, on each trial in stage 1, subjects made an initial choice between two stimuli, which led with fixed probabilities (70 and 30% of choices) to one of two pairs of stimuli in stage 2. Each of the four second-stage stimuli was associated with probabilistic £1 monetary reward (in the reward version of the task) or loss (in the punishment version of the task), with probability varying slowly and independently over time (0.25 to 0.75). The punishment version had a different colour code and stimuli set on the first and second task stages. Both versions of the task had the same transition probabilities and dynamic range of the reward or the punishment probability. Participants completed 201 trials for each task version divided into three sessions. The order of performance of the task versions was counterbalanced and the two versions were separated by at least 1 h.

Before the experiment, all subjects underwent the self-paced computer-based instructions explaining the structure of the task and providing practice examples. Overall, the subjects were instructed to win as much money as they could in the reward version and to avoid monetary loss in the punishment version of the task. Participants were told that they would be paid for the experiment depending on their cumulative performance in both task versions. They were paid a flat amount of £60 at the end of the experiment.

### Behavioural analysis

Before analysis, we applied the arcsin transformation to the non-normally distributed behavioural variables and log transformation to the reaction times that allowed the normalisation of the data, with Shapiro–Wilk test <0.05 for all variables in both groups.

For both versions of the task, we performed two types of analyses: one a factorial analysis of shifting and staying behaviour (which makes few computational assumptions), and the second the fit of a more structured computational model (Supplementary Information 4).

In the factorial analysis, stay probabilities at the first stage (the probability to choose the same stimulus as in the previous trial), transition probability on the previous trial (common (70%) or rare (30%)) and outcome (loss/no loss or reward/no reward) and group (TD or BAL) were entered into three-way mixed-measures ANOVA.

In a computational-fitting analysis, we fit a previously described hybrid model (Supplementary Information 4)^22^ to choice behaviour, estimating free parameters for each subject separately by the method of maximum likelihood. This model contains a separate term for model-free temporal difference algorithm and model-based reinforcement-learning algorithm.

Model selection was performed with a group-level random-effect analysis of the log-evidence obtained for each tested model and subject (Supplementary Information 5). The estimated parameters were fitted to the winning model (see Supplementary Information 5 for parameters optimisation) and were compared between the groups using multivariate ANOVA analysis after normality distribution test and square root transformation of the non-normally distributed variables.

## Results

A total of 22 TD and 22 control (BAL) healthy volunteers were included in the study in a double-blind, counterbalanced design. The groups were matched by gender, age and had no differences in IQ level (Supplementary Table S1).

Post-procedure biochemical analysis showed that TD robustly decreased the TRP:ΣLNAAs ratio relative to the BAL group (main effect of group: *F*_(1,42)_=41.595, *P*<0.0001; main effect of time: *F*_(1,42)_=5.402, *P*=0.025; group × time interaction: F_(1,42)_=41.916, *P*<0.0001). *Post hoc* analysis showed significant (*t*_42_=10.634, *P*<0.0001) reduction of serum TRP concentration in the TD group (mean±s.d.: 75.78±23.07%), but not in BAL (mean±s.d.: 25.00±2.5%, *t*_42_=1.6, *P*=0.18). There was no effect of task order or an interaction of task order with TD (both *F*_(1,42)_<1.0).

We considered staying and shifting of responses as direct markers of model-free and model-based learning. Using mixed-measures ANOVA, we examined the probability of staying or shifting at the first task stage dependent on the between-subjects factor of group (TD or BAL) and within-subject factors of task valence (reward or punishment), outcome (rewarded, non-rewarded, punished or unpunished) and transition probability on the previous trial (common (70%) or rare (30%)).

We found main effects of group (*F*_(1,41)_=4.22, *P*=0.046), outcome (*F*_(1,41)_=17.06, *P*<0.0001) and transition probability (*F*_(1,41)_=32.16, *P*<0.0001), but no main effect of valence (*F*_(1,41)_=1.46, *P*=0.22). Across all subjects and conditions, the finding of both a main effect of outcome and outcome × transition probability interaction (*F*_(1,41)_=28.24, *P*<0.0001) showed that the subjects used both model-free and model-based strategies, respectively. Importantly, the outcome × transition probability interaction (the signature of model-based learning) was significantly modulated by TD (outcome × transition probability × group interaction (*F*_(1,41)_=6.21, *P*=0.017)), and this modulation was itself further modulated by valence (valence × outcome × transition probability × group interaction (*F*_(1,41)_=11.55, *P*=0.001)). There was no outcome × group interaction (*F*_(1,41)_=0.78, *P*=0.38), suggesting an absence of effect of TD on model-free learning.

**Figure 2:**
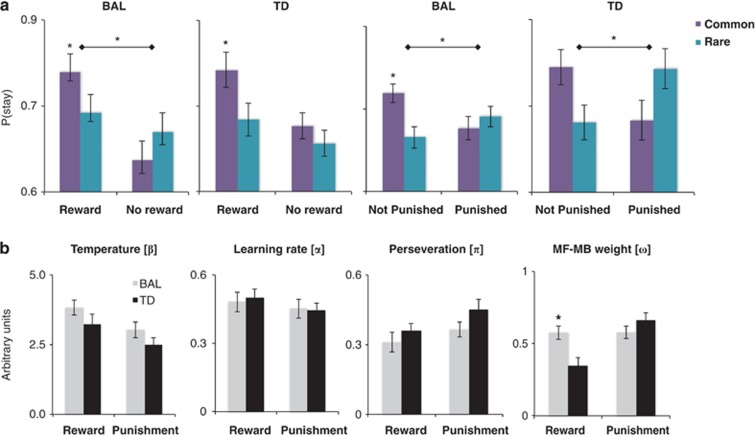
(**a**) Factorial (stay-shift) behavioural results. Separate analysis of task valence showed a mixed choice strategy in BAL and a shift to a model-free choice strategy in the TD group in the reward condition. In the loss condition, the significant interaction between outcome × transition in the TD group indicates a shift of behavioural choice towards a model-based strategy. (**b**) Computationally fitted behavioural results before arscin transformation. Compared with BAL, the TD group showed a significant difference in the weighting factor ω in reward condition. BAL=control group; TD=TRP-depleted group. *P<0.05.

These results indicate that TD affects model-based behaviour in a way that depends on valence, justifying further analyses separated by task valence (reward versus punishment) and by group (TD versus BAL) (Figure 2a).

This analysis of task valence showed a main effect of outcome (i.e., reward or no reward) (*F*_(1,42)_=26.18, *P*<0.0001), transition probability (*F*_(1,42)_=4.87, *P*=0.033) and an outcome × transition probability × group interaction (*F*_(1,42)_=6.63, *P*=0.014) in the reward version. *Post hoc* separate comparisons of BAL versus TD showed a main effect of outcome (*F*_(1,21)_=14.62, *P*=0.001) and an outcome × transition probability interaction (*F*_(1,21)_=6.65, *P*=0.018) in the BAL group only (Figure 2a), indicating both model-free and model-based components in choice performance, in accordance with previous results.^22^ In the TD group, the only significant main effect was that of outcome (*F*_(1,21)_=11.58, *P*=0.003) suggested a behavioural shift to the model-free strategy (Figure 2a).

In the punishment version, there were main effects of transition probability (*F*_(1,42)_=7.88, *P*=0.008) with a significant outcome × transition probability interaction (*F*_(1,42)_=18.80, *P*<0.0001), but no main effect of outcome (i.e., loss or no loss) (*F*_(1,42)_=0.24, *P*=0.62). Overall, this result shows that subjects were aware of the task structure and demonstrated model-based behaviour in this task version. *Post hoc* analysis showed a mixed strategy (both model-free and model-based components in choice performance) in BAL: main effect of outcome (*F*_(1,21)_=8.04, *P*=0.01) and outcome × transition probability interaction (*F*_(1,21)_=4.77, *P*=0.04). For TD, there was only a significant outcome × transition interaction (*F*_(1,21)_=12.07, *P*=0.002), suggesting the use of a model-based strategy in this version of the task (Figure 2a). Overall, these results suggest that TD reduces model-based learning in the reward condition, while promoting it in the punishment condition.

In addition to the preceding factorial analysis of staying-shifting behaviour, we examined these results more closely by fitting participants' choices to a computational model of the learning process, so as to estimate the effects of our manipulations in terms of the parameters of the model, which have interpretations in terms of specific computational processes.^22^ We first used model selection (Supplementary Tables S2 and SI.5) to determine which parameters should be included to optimally model the data. In this analysis, we fitted the behavioural data with computational models of increasing complexity from a pure model-free reinforcement-learning model Q-SARSA (two free parameters) to more complex ‘hybrid' models involving both model-based and model-free learning (four free parameters).

Similar to previous reports,^11^ the model with the best fit for the data in each group of subjects (TD and BAL) and task valence (reward versus punishment) had four free parameters controlling both model-based and model-free learning: learning rate *α*, softmax temperature *β* (control the choice randomness), perseverance index *ρ* (captures perseveration (*ρ*>0) or shifting (*ρ*<0) in the first-stage choices) and the weighting factor *ω*, which provides an index of the relative engagement of a model-free versus model-based behavioural choices (where lower scores indicate a shift to habitual model-free choices and higher scores indicate a shift to model-based choices).

In accordance with data for stay and shift behaviour in the reward condition, a multivariate ANOVA showed that, compared with the BAL group, the TD group had a lower *ω* (*F*_(1,39)_=6.93, *P*=0.012) and a trend to a higher perseverance index (*F*_(1,37)_=2.99, *P*=0.092). In contrast, there was no significant difference between the groups in the parameters of the loss version of the task (see also Supplementary Table 4, Figure 2b and Supplementary Information 6).

The analysis of choice reaction times showed no difference between the groups on the first or second stages of the task (all *P*>0.1) or between loss and reward version of the task (all *P*>0.1). There were more omitted trials in the punishment version of the task in both groups, but no difference between the groups (*F*<1.0). Finally, there was no difference between the groups in cumulative learning in both versions of tasks (reward: *F*_(1,42)_<0.1, loss: *F*_(1,42)_=1.31, *P*=0.25).

## Discussion

The balance between model-based and model-free behavioural control is suggested to determine at least some aspects of our decisional process, being framed as a competition and/or co-operation between a flexible prospective goal-directed system and fixed retrospective system.^4^

Here, we investigated the modulatory role of serotonin in the balance between these two systems, and provide evidence that diminished serotonin neurotransmission, effected by TD, influences goal-directed behaviour while leaving intact the model-free choice strategy. Overall, in the reward condition, TD impaired goal-directed behaviour and shifted the balance towards the model-free strategy. However, this effect changed with motivational valence. In the punishment condition, the factorial analysis pointed to an increase of behavioural goal-directness, although a secondary computational model-fitting analysis failed to fully corroborate this second result. Both animal^23^ and human studies^26^ have suggested a selective TD effect on central serotonin, with no effect on DA and norepinephrine neurotransmission; hence, these findings are likely to be neurochemically specific.

These effects of TD support a dual role for 5-HT mechanisms in the choice strategy balance depending on outcome valence. Modulation of the representation of average reward rate is a possible mechanism of shifting of the behavioural balance in either reward or punishment conditions. This interpretation grows out of several ideas from the modelling literature: first, that serotonin may help report average reward^27, 28^ and, second, that this quantity should affect the tendency to use model-based choice, as it represents the opportunity cost (or in the punishment condition, benefit) of time spent deliberating.^29, 30^ More specifically, in the ‘average-case' reinforcement-learning model, the average reward is a signal that provides an estimation of the overall ‘goodness' or ‘badness' of the environment^27^ (also see the Supplementary Information 6 for further discussion on this point).

A tonic serotonergic signal has been previously suggested to report average reward rate over long sequences of trials, either positively^27^ or negatively.^28^ Lowering serotonin neurotransmission in the brain via the TD procedure would result in increases in the average reward signal representation and a shift toward model-free responding. The opportunity cost considerations of Keramati *et al*^30^ offer an explanation of the effect of TD on the reward condition. Finally, as for the punishment condition, the opportunity cost of time inverts and becomes a benefit (as any time spent not being punished is better than average^31^), which may help explain why the sign of the effect reverses in this condition (also see the Supplementary Information 6 for further discussion on this point).

One can also argue that the effects observed here might ultimately result from a nonspecific 5-HT depletion effect on cognitive functions that affects performance on the two-stage task. Indeed, there are quite consistent deleterious effects of 5-HT depletion on working memory,^32^ which may prevent engagement in model-based strategies.^33^ However, in that case, we would have expected promotion of model-free behavioural choice, independent of valence, but that result was not observed here.

Confidence or uncertainty about the choice at different levels (i.e., confidence about reward outcome or higher level confidence about that belief) could also potentially affect results. Numerous studies have shown the main effect of uncertainty to be on the modulation of learning rates.^34, 35^ However, as we did not observe any difference in learning rates between the groups in either valence conditions, it is also unlikely that effects on choice confidence could explain the reported results.

Low serotonin has been also showed to prone the risky decisions in reward condition^36, 37^ and risk-aversion under the punishment.^38^ However, how the risk influences the goal-directed behaviours remains unclear, and further studies are needed to address this point.

Finally, in view of the proposed functional interaction of 5-HT with brain DA and evidence for the influence of DA in the balance between model-based and model-free strategies, it is possible that the effect of TD was mediated ultimately via interactions with the DA system. TD had the opposite effect to that of levodopa administration^11^ by alteration of the model-based strategy. This would argue for synergy or co-operation between the DA and 5-HT systems. Nonetheless, a recent study has shown highly parallel effects of selective 5-HT depletion in rats and TD in humans on a similar task measuring increases in impulsive behaviour,^39^ suggesting that the effects of TD are likely to be mediated via 5-HT loss. However, our results will ultimately require confirmation using other means to reduce central 5-HT function,^40^ although there are no other clear-cut means to do this in human volunteers. The effects of nonspecific 5-HT receptor agents, for example, would be difficult to interpret. However, it would be of theoretical, as well as clinical, value to test the effects of enhanced 5-HT neurotransmission produced by administration of selective serotonin re-uptake inhibitors. In addition, there are no available data to clarify how DA modulates behavioural choice in the punishment condition of the task and therefore the nature of any possible interaction with the 5-HT system. However, it has been reported following either DA D2 receptor blockade or Parkinson's disease, which is characterised by diminished striatal DA neurotransmission, that there is greater attention to stimuli associated with punishment than with reward.^8, 9, 41^ We also did not show a specific effect of 5-HT depletion on model-free or habitual response in the behavioural analysis. The two-step task or model-free reinforcement learning has been suggested not to fully capture habit expression; further studies focusing on conventional over-training and testing in extinction may help clarify the effect of 5-HT depletion on habit.^5^

The major implication of this study is that 5-HT contributes to both appetitive and aversive learning, an increasingly supported view.^13, 42, 43^ As model-free and model-based learning appear to have different anatomical correlates within the corticostriatal circuitry, as shown by functional neuroimaging^44, 45, 46^ and rodent lesion studies,^1, 47^ it could be speculated that decreases in central 5-HT neurotransmission may affect these types of learning at different anatomical locations.

Finally, our findings are also of clinical interest, as impairment of goal-directed responses has been put forward as a theoretical framework for a range of psychiatric disorders.^48^ In particular, impairment of goal-directed behavioural control has been evidenced in obsessive-compulsive disorders, as well as in substance addictions and eating disorders.^49, 50, 51^

## References

[bib1] Balleine BW, O'Doherty JP. Human and rodent homologies in action control: corticostriatal determinants of goal-directed and habitual action. Neuropsychopharmacology 2010; 35: 48–69. 19776734 10.1038/npp.2009.131 PMC3055420

[bib2] Dickinson A. Actions and habits: the development of behavioural and autonomy. Philos Trans R Soc Lond B Biol Sci 1985; 308: 67–78.

[bib3] Dolan RJ, Dayan P. Goals and habits in the brain. Neuron 2013; 80: 312–325. 24139036 10.1016/j.neuron.2013.09.007 PMC3807793

[bib4] Daw ND, Niv Y, Dayan P. Uncertainty-based competition between prefrontal and dorsolateral striatal systems for behavioral control. Nat Neurosci 2005; 8: 1704–1711. 16286932 10.1038/nn1560

[bib5] Dezfouli A, Lingawi NW, Balleine BW. Habits as action sequences: hierarchical action control and changes in outcome value. Philos Trans R Soc Lond B Biol Sci 2014; 369; doi:10.1098/rstb.2013.0482. 10.1098/rstb.2013.0482 PMC4186235 25267824

[bib6] Wunderlich K, Dayan P, Dolan RJ. Mapping value based planning and extensively trained choice in the human brain. Nat Neurosci 2012; 15: 786–791. 22406551 10.1038/nn.3068 PMC3378641

[bib7] Smittenaar P, Fitzgerald TH, Romei V, Wright ND, Dolan RJ. Disruption of dorsolateral prefrontal cortex decreases model-based in favor of model-free control in humans. Neuron 2013; 80: 914–919. 24206669 10.1016/j.neuron.2013.08.009 PMC3893454

[bib8] Frank MJ, Seeberger LC, O'Reilly RC. By carrot or by stick: cognitive reinforcement learning in parkinsonism. Science 2004; 306: 1940–1943. 15528409 10.1126/science.1102941

[bib9] Pessiglione M, Seymour B, Flandin G, Dolan RJ, Frith CD. Dopamine-dependent prediction errors underpin reward-seeking behaviour in humans. Nature 2006; 442: 1042–1045. 16929307 10.1038/nature05051 PMC2636869

[bib10] Worbe Y, Palminteri S, Hartmann A, Vidailhet M, Lehericy S, Pessiglione M. Reinforcement learning and gilles de la tourette syndrome: dissociation of clinical phenotypes and pharmacological treatments. Arch Gen Psychiatry 2011; 68: 1257–1266. 22147843 10.1001/archgenpsychiatry.2011.137

[bib11] Wunderlich K, Smittenaar P, Dolan RJ. Dopamine enhances model-based over model-free choice behavior. Neuron 2012; 75: 418–424. 22884326 10.1016/j.neuron.2012.03.042 PMC3417237

[bib12] Boureau YL, Dayan P. Opponency revisited: competition and cooperation between dopamine and serotonin. Neuropsychopharmacology 2011; 36: 74–97. 20881948 10.1038/npp.2010.151 PMC3055522

[bib13] Dayan P, Huys QJ. Serotonin in affective control. Annu Rev Neurosci 2009; 32: 95–126. 19400722 10.1146/annurev.neuro.051508.135607

[bib14] Palminteri S, Clair AH, Mallet L, Pessiglione M. Similar improvement of reward and punishment learning by serotonin reuptake inhibitors in obsessive-compulsive disorder. Biol Psychiatry 2012; 72: 244–250. 22325972 10.1016/j.biopsych.2011.12.028

[bib15] Miyazaki KW, Miyazaki K, Doya K. Activation of dorsal raphe serotonin neurons is necessary for waiting for delayed rewards. J Neurosci 2012; 32: 10451–10457. 22855794 10.1523/JNEUROSCI.0915-12.2012 PMC6621383

[bib16] Miyazaki KW, Miyazaki K, Tanaka KF, Yamanaka A, Takahashi A, Tabuchi S et al. Optogenetic activation of dorsal raphe serotonin neurons enhances patience for future rewards. Curr Biol 2014; 24: 2033–2040. 25155504 10.1016/j.cub.2014.07.041

[bib17] Schweighofer N, Bertin M, Shishida K, Okamoto Y, Tanaka SC, Yamawaki S et al. Low-serotonin levels increase delayed reward discounting in humans. J Neurosci 2008; 28: 4528–4532. 18434531 10.1523/JNEUROSCI.4982-07.2008 PMC6670945

[bib18] den Ouden HE, Swart JC, Schmidt K, Fekkes D, Geurts DE, Cools R. Acute serotonin depletion releases motivated inhibition of response vigour. Psychopharmacology (Berl) 2014; 232: 1303–1312. 25326051 10.1007/s00213-014-3762-4

[bib19] den Ouden HE, Daw ND, Fernandez G, Elshout JA, Rijpkema M, Hoogman M et al. Dissociable effects of dopamine and serotonin on reversal learning. Neuron 2013; 80: 1090–1100. 24267657 10.1016/j.neuron.2013.08.030

[bib20] Crockett MJ, Clark L, Robbins TW. Reconciling the role of serotonin in behavioral inhibition and aversion: acute tryptophan depletion abolishes punishment-induced inhibition in humans. J Neurosci 2009; 29: 11993–11999. 19776285 10.1523/JNEUROSCI.2513-09.2009 PMC2775933

[bib21] Geurts DE, Huys QJ, den Ouden HE, Cools R. Serotonin and aversive Pavlovian control of instrumental behavior in humans. J Neurosci 2013; 33: 18932–18939. 24285898 10.1523/JNEUROSCI.2749-13.2013 PMC6618702

[bib22] Daw ND, Gershman SJ, Seymour B, Dayan P, Dolan RJ. Model-based influences on humans' choices and striatal prediction errors. Neuron 2011; 69: 1204–1215. 21435563 10.1016/j.neuron.2011.02.027 PMC3077926

[bib23] Ardis TC, Cahir M, Elliott JJ, Bell R, Reynolds GP, Cooper SJ. Effect of acute tryptophan depletion on noradrenaline and dopamine in the rat brain. J Psychopharmacol 2009; 23: 51–55. 18562433 10.1177/0269881108089597

[bib24] Biggio G, Fadda F, Fanni P, Tagliamonte A, Gessa GL. Rapid depletion of serum tryptophan, brain tryptophan, serotonin and 5-hydroxyindoleacetic acid by a tryptophan-free diet. Life Sci 1974; 14: 1321–1329. 4823644 10.1016/0024-3205(74)90440-8

[bib25] Carpenter LL, Anderson GM, Pelton GH, Gudin JA, Kirwin PD, Price LH et al. Tryptophan depletion during continuous CSF sampling in healthy human subjects. Neuropsychopharmacology 1998; 19: 26–35. 9608574 10.1016/S0893-133X(97)00198-X

[bib26] Cox SM, Benkelfat C, Dagher A, Delaney JS, Durand F, Kolivakis T et al. Effects of lowerd serotonin transmission on cocaine-induced striatal dopamine response: PET (11C)raclopride study in humans. Br J Psychiatry 2011; 199: 391–397. 21543823 10.1192/bjp.bp.110.084178

[bib27] Daw N, Kakadeb S, Dayan P. Opponent interactions between serotonin and dopamine. Neural Networks 2002; 15: 603–616. 12371515 10.1016/s0893-6080(02)00052-7

[bib28] Cools R, Nakamura K, Daw ND. Serotonin and dopamine: unifying affective, activational, and decision functions. Neuropsychopharmacology 2011; 36: 98–113. 20736991 10.1038/npp.2010.121 PMC3055512

[bib29] Niv Y, Daw ND, Joel D, Dayan P. Tonic dopamine: opportunity coast and the control of response vigor. Psychopharmacology 2007; 191: 507–520. 17031711 10.1007/s00213-006-0502-4

[bib30] Keramati M, Dezfouli A, Piray P. Speed/accuracy trade-off between the habitual and the goal-directed process. PLoS Comput Biol 2011; 7: e1002055. 21637741 10.1371/journal.pcbi.1002055 PMC3102758

[bib31] Dayan P. Instrumental vigor in punishment and reward. Eur J Neurosci 2012; 35: 1152–1168. 22487044 10.1111/j.1460-9568.2012.08026.x

[bib32] Cowen P, Sherwood AC. The role of serotonin in cognitive function: evidence from recent studies and implications for understanding depression. J Psychopharmacol 2013; 27: 575–583. 23535352 10.1177/0269881113482531

[bib33] Otto AR, Raiob CM, Chiangb A, Phelpsa EA, Daw ND. Working-memory capacity protects model-based learning from stress. PNAS 2013; 110: 20941–20946. 24324166 10.1073/pnas.1312011110 PMC3876216

[bib34] Courville AC, Daw N, Touretzk DS. Bayesian theories of conditioning in a changing world. Trends Cogn Sci 2006; 10: 294–300. 16793323 10.1016/j.tics.2006.05.004

[bib35] Behrens TE, Woolrich MW, Walton ME, Rushworth MF. Learning the value of information in an uncertain world. Nat Neurosci 2007; 10: 1214–1221. 17676057 10.1038/nn1954

[bib36] Koot S, Zoratto F, Cassano T, Colangeli R, Laviola G, van den Bos R et al. Compromised decision-making and increased gambling proneness following dietary serotonin depletion in rats. Neuropharmacology 2012; 62: 1640–1650. 22118880 10.1016/j.neuropharm.2011.11.002

[bib37] Long AB, Kuhn CM, Platt ML. Serotonin shapes risky decision making in monkeys. Soc Cogn Affect Neurosci 2009; 4: 346–356. 19553236 10.1093/scan/nsp020 PMC2799948

[bib38] Macoveanu J, Rowe JB, Hornboll B, Elliott R, Paulson OB, Knudsen GM et al. Playing it safe but losing anyway—serotonergic signaling of negative outcomes in dorsomedial prefrontal cortex in the context of risk-aversion. Eur Neuropsychopharmacol 2013; 23: 919–930. 23051938 10.1016/j.euroneuro.2012.09.006 PMC4606974

[bib39] Worbe Y, Savulich G, Voon V, Fernandez-Egea E, Robbins TW. Serotonin depletion induces ‘waiting impulsivity' on the human four choice serial reaction time task: cross-species translational significance. Neuropsychopharmacology 2014; 39: 1519–1526. 24385133 10.1038/npp.2013.351 PMC3988556

[bib40] Crockett MJ, Clark L, Roiser JP, Robinson OJ, Cools R, Chase HW et al. Converging evidence for central 5-HT effects in acute tryptophan depletion. Mol Psychiatry 2012; 17: 121–123. 21876544 10.1038/mp.2011.106

[bib41] Palminteri S, Lebreton M, Worbe Y, Grabli D, Hartmann A, Pessiglione M. Pharmacological modulation of subliminal learning in Parkinson's and Tourette's syndromes. Proc Natl Acad Sci USA 2009; 106: 19179–19184. 19850878 10.1073/pnas.0904035106 PMC2776465

[bib42] McCabe C, Mishor Z, Cowen PJ, Harmer CJ. Diminished neural processing of aversive and rewarding stimuli during selective serotonin reuptake inhibitor treatment. Biol Psychiatry 2010; 67: 439–445. 20034615 10.1016/j.biopsych.2009.11.001 PMC2828549

[bib43] Seymour B, Daw ND, Roiser JD, Dayan P, Dolan R. Serotonin selectively modulates reward value in human decision-making. J Neurosci 2012; 31: 5833–5842. 10.1523/JNEUROSCI.0053-12.2012 PMC5321452 22539845

[bib44] Tricomi EM, Balleine BW, O'Doherty JP. A specific role for posterior dorsolateral striatum in human habit learning. Eur J Neurosci 2009; 29: 2225–2232. 19490086 10.1111/j.1460-9568.2009.06796.x PMC2758609

[bib45] Valentin VV, Dickinson A, O'Doherty JP. Determining the neural substrates of goal-directed learning in the human brain. J Neurosci 2007; 27: 4019–4026. 17428979 10.1523/JNEUROSCI.0564-07.2007 PMC6672546

[bib46] Gläscher J, Daw N, Dayan P, O'Doherty J. States versus rewards: dissociable neural prediction error signals underlying model-based and model-free reinforcement learning. Neuron 2010; 66: 585–595. 20510862 10.1016/j.neuron.2010.04.016 PMC2895323

[bib47] Killcross S, Coutoureau E. Coordination of action and habits in the medial prefrontal cortex of rats. Cereb Cortex 2003; 13: 400–408. 12631569 10.1093/cercor/13.4.400

[bib48] Griffiths KR, Morris RW, Balleine BW. Translatinal studies of goal-directed action as a framework for classifying deficit across psychiatric disorders. Front Syst Neurosci 2014; 8: 101. 24904322 10.3389/fnsys.2014.00101 PMC4033402

[bib49] Gillan CM, Robbins TW. Goal-directed learning and obsessive-compulsive disorders. Philos Trans R Soc Lond B Biol Sci 2014; 369560 doi:10.1098/rstb.2013.0475. 10.1098/rstb.2013.0475 PMC4186229 25267818

[bib50] Gillan CM, Papmeyer M, Morein-Zamir S, Sahakian BJ, Fineberg NA, Robbins TW et al. Disruption in the balance between goal-directed behavior and habit learning in obsessive-compulsive disorder. Am J Psychiatry 2011; 168: 718–726. 21572165 10.1176/appi.ajp.2011.10071062 PMC3533260

[bib51] Voon V, Derbyshire K, Rück C, Irvine MA, Worbe Y, Enander J et al. Disorders of compulsivity: a common bias towards learning habits. Mol Psychiatry 2014; 20: 345–352. 24840709 10.1038/mp.2014.44 PMC4351889

