# Supplementary material for: Valence-dependent influence of serotonin depletion on model-based choice strategy
Source: Mol Psychiatry. 2015 Apr 14;21(5):624–9. doi: 10.1038/mp.2015.46 (PMC4519524; doi:10.1038/mp.2015.46)
Supplement: Supplementary Information [file mp201546x1.docx]

**Supplementary Information**

**Valence-dependent influence of serotonin depletion on model-based choice strategy**

Worbe Y, Palminteri S, Savulich G, Daw N.D, Fernandez-Egea E, Robbins T.W, Voon V

**SI 1. Participants’ inclusion criteria and characteristics**

The East of England-Essex Research Ethics Committee approved this study. Participants were recruited from university-based advertisements and from Cambridge BioResource (www.cambridgebioresource.org.uk) and gave informed consent prior to participation. The inclusion criteria were as follows: age 18 – 45 years, no history of neurological or psychiatric disorders as assessed with the Mini International Neuropsychiatric Inventory ([1](#_ENREF_1)), no regular or recreational use of drugs including nicotine, no significant physical illness and not currently taking any type of regular medication (except contraceptive pills for women).

**Supplementary Table 1. Participants’ demographic and behavioural data**

|  | **BAL (n=22)** | **TD (n=22)** | **t** | **p** |
| --- | --- | --- | --- | --- |
| Age | 27.78 ± 1.61 | 30.50 ± 1.84 | 0.139 | 0.890 |
| Men (number) | 11 | 10 | 0.091* | 0.762* |
| IQ | 120.67 ± 1.96 | 120.20 ± 1.62 | 0.037 | 0.879 |
| BDI | 3.54 ± 0.92 | 3.05 ± 0.79 | 0.413 | 0.682 |
| STAI, trait | 46.84 ± 1.30 | 44.88 ± 0.10 | -0.975 | 0.335 |
| STAI, state | 44.23 ± 1.04 | 45.62 ± 0.97 | -1.264 | 0.213 |
| STAI, state, post-procedure | 41.26 ± 1.26 | 44.33 ± 1.09 | -1.64 | 0.293 |

*- chi square test; Reported as means ± SEM values, TD = tryptophan depleted group; BAL = control group. BDI = Beck Depression Inventory; STAI = Spielberg State-Trait Anxiety Inventory (STAI).

**SI. 2 Session schedule and** **tryptophan depletion procedure**

Participants were assigned to receive either the tryptophan depleting drink (TD) or the placebo mixture in a randomized, placebo-controlled, double blind order.

Prior to participation, participants were asked to abstain from food and alcohol 12 hours before the testing session. Upon arrival in the morning, they completed questionnaires, gave a blood sample for the biochemical measures and ingested either the placebo or the TD drink. To control for mood and anxiety state, we administrated the Beck Depression Inventory (BDI) ([2](#_ENREF_2)) and the Spielberg State-Trait Anxiety Inventory (STAI) ([3](#_ENREF_3)). A proxy of an intelligence quotient (IQ) was measured using the National Adult reading Test (NART) ([4](#_ENREF_4)). To ensure stable and low tryptophan levels, the behavioral testing was performed and the second blood sample taken after a resting period of approximately 5 hours. Low-protein snacks (biscuits, vegetable sandwiches and fruits) were provided to the participants during the waiting period.

In the TD procedure, tryptophan was depleted by ingestion of a liquid amino acid load that did not contain tryptophan but did include other large neutral amino acids (LNAAs). Amino acid mixtures (SHS International, Liverpool, UK) were as follows: TD: L-alanine, 4.1 g; L-arginine, 3.7 g; L-cystine, 2.0 g; glycine, 2.4 g; L-histidine, 2.4 g; L-isoleucine, 6 g; Lleucine, 10.1 g; L-lysine, 6.7 g; L-methionine, 2.3 g; Lproline, 9.2 g; L-phenylalanine, 4.3 g; L-serine, 5.2 g; Lthreonine, 4.9 g; L-tyrosine, 5.2 g; and L-valine, 6.7 g. Total: 75.2 g. Placebo: same as ATD, plus 3.0 g of L-tryptophan. Total: 78.2 g.

The drinks were prepared by stirring the mixture and lemon–lime flavouring into 200 ml tap water.

Plasma total amino acid concentrations (tyrosine, valine, phenylalanine, isoleucine, leucine, and tryptophan) were measured by means of high-performance liquid chromatography with fluorescence end-point detection and precolumn sample derivatization. The tryptophan/large neutral amino acid (TRP:ΣLNAAs) ratio was calculated as an indicator of central serotonergic function. The obtained values were entered in repeated measures ANOVA with time as a dependent and group as independent factors.

**SI 3. Task**

We used the two-stage decision task with reward and punishment conditions implemented by MATLAB 2010a and Cogent 2000.

The reward version of task was identical to previously published task by Daw et al. (2011). The punishment version had a different colour code and stimuli set on the first and second task stages. Both versions of the task had the same transition probabilities and dynamic range of the reward or the punishment.

Before the experiment, all subjects underwent the self-paced (approximately 10 min) computer-based instructions explaining the structure of the task and providing practice examples. Overall, the subjects were instructed to win as much money as they could in the reward version and to avoid monetary loss in the punishment version of the task. The order of performance of the versions of the task was counter-balanced and the two versions were separated by at least 1 hour.

On each trial in Stage 1, subjects made an initial choice between two stimuli, which led with fixed probabilities (70 % and 30 % of choices) to one of two pairs of stimuli in Stage 2. Each of the four second-stage stimuli was associated with a probabilistic monetary reward (in the Reward version of the task) or loss (in the Punishment version of the task), with probability varying slowly and independently over time (0.25 to 0.75), as shown in Fig 1A.

On each stage of the task, participants had 2 seconds to make a decision. The transition time between the stages was 1.5 seconds. If no response was performed within 2 seconds, the trial was aborted (indicated by red cross on the stimuli). No outcome was associated with omitted trials. The reward was a picture of a one-pound coin. The punishment was a monetary loss of £1, indicated as a one pound coin overlaid with a red cross. Participants completed 201 trials for each task version divided into 3 sessions (with a mean duration of session of approximately 9 minutes). The omitted trials were discarded from the analysis for each task version and for each participant.

Participants were told that they would be paid for the experiment depending on their cumulative performance in both task versions. They were paid a flat amount of £60 at the end of the experiment.

The mean cumulative earnings for both groups were as follows: (Mean ± SD), Reward: TD - 28.21 ± 0.96 £; BAL - 28.50 ± 0.99 £; Loss: TD - 25.50 ± 1.06 £; BAL - 27.59 ± 0.85 £.

The task was a part of a larger tests battery and was generally performed as the 1^st^ and the 3d task in the battery order. The mean time between the drink intake and task performance was respectively 5 and 6.5 hours.

**SI 4. Computational model**

The detailed description of the hybrid model is provided in Daw et al. (2011). The algorithm included both model-based and model-free subcomponents, which allowed for mapping each state-action pair to its expected future value.

The model-free strategy was computed using the SARSA (λ) temporal difference learning. At each stage *i* of each trial *t,* the value for the each state-action pair was calculated as follows:

*Q_TD_ (s_i,t_,a_i,t_) = Q_TD_ (s_i,t_,a_i,t_) + α_i_ δ_i,t_*

where *δ_i,t_ = r_i,t_ + Q_TD_ (s_i+1,t_ , a_i + 1,t_) - Q_TD_ (s_i,t_ ,a_i,t_)* and *α_i_*  is a free learning parameter. The full model allows different learning rates *α_1_* and *α_2_* for the two task stages. The reinforcement eligibility parameter (λ) determines the update of the first-stage action by the second-stage prediction error as follows: *Q_TD_ (s_1,t_ , a_i1,t_) = Q_TD_ (s_1,t_ ,a_1,t_) + a_1_λδ_2,t ._*

The model-based reinforcement-learning algorithm was computed by mapping state-action pairs to a transition function and assuming that participants choose between two possibilities, as follows: *P(S_B_ ⏐S_A_, a_A_) = 0.7, P(S_C_ ⏐S_A_, a_B_) = 0.7* for the common or *P(S_B_ ⏐S_A_, a_A_) = 0.3 P(S_C_ ⏐S_A_, a_B_) = 0.3* for the rare transition, where S is a state (first stage: S_A_; second stage: S_B_ and S_C_), and a is an action (two actions - a_A_ and a_B_).

The action value (Q_MB_) was computed at each trial from the estimates of the transition probabilities and rewards and was defined for the first stage as follows:

*Q_MB_ (s_A_,a_i_) = P(s_B_|s_A_,a_i,_) max_a_ Q_TD_(s_B_,a) + P(s_C_|s_A_,a_i,_) max_a_ Q_TD_(s_C_,a)*

Finally, to connect the values to choices, the weighted sum of the model-free and model-based values was computed for the first stage as defined:

*Q_net_(s_A_,a_j_) = w Q_MB_(s_A_,a_j_) + (1-w) Q_TD_(s_A_,a_j_)*

where *w* is the weighting parameter.

Assuming that two approaches coincide at the second stage, and that Q_MB_ = Q_TD_, at the second stage Q_net_= Q_MB_ = Q_TD._ Then, the probability of a choice is the softmax equation for

Q_net_:

*P(a_i,t_ = a|s_i,t_) = exp (β_i_[Q_net_(s_i,t_,a) + p * rep(a)])*

_____________________________

*Σ_a’_ exp (β_i_[Q_net_(s_i,t_,a) + p * rep(a)])*

where the free inverse temperature parameters (β_i_) control the choice randomness, and *p* captures perseveration (p > 0) or switching (p < 0) in the first-stage choices. In total, the fully parameterized model contains 7 free parameters (β1, β2, α1, α2, λ, π, **ω**), with special cases of pure model-based (**ω** = 1) and model-free (**ω** = 0) models.

**SI 5. Parameters optimization and model selection procedure.**

We optimized model parameters by maximizing the Laplace approximation to the model evidence with Matlab’s fmincon function. To ensure convergence the number of function iterations and evaluation of fmincon function were increased from the default value to 1000 000. The Laplace approximation to the model evidence (log of posterior probability) was calculated as: LPP = log(∑P(D|M,θ))),

where D, M and θ represent the data, model and model parameters respectively, assuming the parameters distributed as follows: learning rate betapdf(lr1,1.1,1.1), temperature gampdf(beta1,1.2,5), perseveration normpdf(ps,0,1) and finally model-free/model-based weighting parameter betapdf(w,1.1,1.1). The same approach has been used in a previous study in (Daw et al Neuron 2011).

The probability corresponds to the marginal likelihood, which is the integral over the parameter space of the model likelihood weighted by the prior on free parameters. This probability increases with the likelihood (which measures the accuracy of the fit) and is penalized by the integration over the parameter space (which measures the complexity of the model). The model evidence thus represents a trade-off between accuracy and complexity and can guide model selection.

Model selection was performed with a group-level random-effect analysis of the log-evidence obtained for each model and subject, using the VB-toolbox ([5](#_ENREF_5)) (<https://code.google.com/p/mbb-vb-toolbox/>). This procedure estimates the expected frequencies of the model (denoted PP) and the exceedance probability (denoted XP) for each model within a set of models, given the data gathered from all subjects. Expected frequency quantifies the posterior probability, i.e. the probability that the model generated the data for any randomly selected subject. This quantity must be compared to chance level (one over the number of models in the search space). Exceedance probability quantifies the belief that the model is more likely than all the other models of the set, or in other words, the confidence in the model having the highest expected frequency.

**SI 6. Parameters correlation.**

Across all subjects, we found the following correlations of model parameters that survived Bonferroni correction of multiple comparisons: in the Loss version of the task - a significant correlation between **β** and **π** (r = -0.482, p = 0.002) and in the Reward version of the task - between **α** and **β** (r = -0.420, p = 0.006).

**SI 7 Supplementary discussion**

Here, we investigated the modulatory role of serotonin in the balance between these two systems and provide evidence that diminished serotonin neurotransmission, effected by TD, influences goal-directed behaviour while leaving intact the model-free choice strategy. Overall, in the reward condition TD impaired goal-directed behaviour and shifted the balance towards the model-free strategy. However, this effect changed with motivational valence. In the punishment condition, the factorial analysis pointed to an increase of behavioural goal-directness, although a secondary computational model-fitting analysis failed to fully corroborate this second result. Both animal ([6](#_ENREF_6)) and human studies ([7](#_ENREF_7)) have suggested a selective TD effect on central serotonin, with no effect on dopamine and norepinephrine neurotransmission, hence these findings are likely to be neurochemically specific.

These effects of TD support a dual role for 5-HT mechanisms in the choice strategy balance depending on outcome valence. Modulation of the representation of average reward rate is a possible mechanism of shifting of the behavioural balance in either reward or punishment conditions. This interpretation grows out of several ideas from the modelling literature: first, that serotonin may help to report average reward ([8](#_ENREF_8), [9](#_ENREF_9)) and second, that this quantity should affect the tendency to employ model-based choice since it represents the opportunity cost (or in the punishment condition, benefit) of time spent deliberating ([10](#_ENREF_10), [11](#_ENREF_11)). More specifically, in the ‘average-case’ reinforcement-learning model, the average reward is a signal that provides an estimation of the overall ‘goodness’ or ‘badness’ of the environment ([8](#_ENREF_8)). Theoretically, this quantity plays an important role in numerous aspects of choice; for instance, it characterizes the opportunity cost of time ([9](#_ENREF_9), [10](#_ENREF_10)): if the average reward is high, then any time spent *not* earning reward is relatively more costly. This opportunity cost effect might affect the balance between model-based and model-free behaviour ([11](#_ENREF_11)). If the brain allocates time to deliberating (to produce model-based behaviour) by balancing the opportunity cost of time spent this way against the rewards gained (by making improved decisions) from doing so, then when the average reward is high, model-free responding is more favored ([11](#_ENREF_11)).

Meanwhile, a tonic serotonergic signal has been previously suggested to report average reward rate over long sequences of trials, either positively ([8](#_ENREF_8)) or negatively ([9](#_ENREF_9)). Putting these two points together, then, on the latter suggestion, lowering serotonin neurotransmission in the brain via the TD procedure would result in increases in the average reward signal representation and a shift toward model-free responding. Keramati et al’s ([11](#_ENREF_11)) opportunity cost considerations thus offer an explanation of the effect of TD on the reward condition. Finally, as for the punishment condition, the opportunity cost of time inverts and becomes a benefit (since any time spent not being punished is better than average ([12](#_ENREF_12))), which may help to explain why the sign of the effect reverses in this condition (please, also see the SI 6 for further discussion on this point).

A related interpretation of the results reported here might be that TD differentially affects the impact or desirability of the rewards or punishments themselves: again, by Keramati’s ([11](#_ENREF_11)) logic, model-based deliberation will be more (or less) worth engaging in the better (or worse) is the outcome thus obtained. Note that in the current model, condition-wise changes in the scaling of rewards should be reflected in changes in the estimated temperature parameter β; in other words, choices should become more or less deterministic. We did not see any such effects. However, this prediction is specific to the softmax choice rule assumed here, and would not be expected under other plausible forms like a Luce choice rule.

**Supplementary Table 2. Values of the best model parameters in all subjects.**

| **Name** | **α** | **β** | **π** | **ω** |
| --- | --- | --- | --- | --- |
| Lower limit | 0 | 0 | -Inf | 0 |
| Upper limit | 1 | Inf | Inf | 1 |
| Mean | 3.2314 | 0.4331 | 0.4447* | 0.5062 |
| Median | 2.7235 | 0.4614 | 0.3434 | 0.5304 |
| Max | 10.8778 | 0.9693 | 1.6741 | 0.9831 |
| Mean | 0.4281 | 0.0004 | 0.0089 | 0.0064 |

Mean ± SEM. *t(87)=11.4, p < 0.001.

**Supplementary Table 3: Model selection results**

|  | **Model 1** | **Model 2** | **Model 3** | **Model 4** |
| --- | --- | --- | --- | --- |
| **DF** | **2 (α, β)** | **3 (α, β, ω)** | **3 (α, β, π)** | **3 (α, β, π , ω)** |
| **All subjects (XP)** | 0 | 0 | 0 | 1 |
| **All subjects (EF)** | 0.0009±0.0003 | 0.0015±0.0005 | 0.0069±0.0007 | **0.9906±0.0011** |
| **Bal/Rew (XP)** | 0 | 0 | 0 | **1** |
| **Bal/Rew (EF)** | 0.0031±0.0023 | 0.0060±0.0036 | 0.0164±0.0039 | **0.9745±0.0073** |
| **Bal/Pun (XP)** | 0 | 0 | 0 | **1** |
| **Bal/Pun (EF)** | 0.0024±0.0022 | 0.0031±0.0028 | 0.0153±0.0046 | **0.9791±0.0073** |
| **TD/Rew (XP)** | 0 | 0 | 0 | **1** |
| **TD/Rew (EF)** | 0.0051±0.0030 | 0.0086±0.0042 | **0.0569±0.0081*^✝^** | **0.9294±0.0104*^✝^** |
| **TD/Pun (XP)** | 0 | 0 | 0 | **1** |
| **TD/Pun (EF)** | 0.0029±0.0024 | 0.0047±0.0031 | 0.0309±0.0057 | **0.9615±0.0084** |

DF - degree of freedom; XP - model exceedance probability; EF - model expected frequency; Bal – control group; TD - tryptophan depleted group. Expected frequencies are reported as Mean ± SEM. * t(21) > 2.9, p < 0.01, paired t-test compared to the TD punishment task. ✝ - t(42) > 3.5, p <0.01, unpaired t-test compared to the Bal reward task.

**Supplementary Table 4. Parameters Values of the best-fitting computational model before arscin transformation**

| **Parameter** | **TD group** | **BAL group** |
| --- | --- | --- |
| **Reward condition :** | | |
| **Learning rate (α)** | 0.48 ± 0.05 | 0.49 ± 0.04 |
| **Choice temperature (β)** | 3.23 ± 0.32 | 3.80 ± 0.44 |
| **Perseverance index (π)** | 0.36 ± 0.04 | 0.31 ± 0.03 |
| **Weighting factor (ω)** | 0.34 ± 0.05 | 0.57 ± 0.06 |
| **Punishment condition :** | | |
| **Learning rate (α)** | 0.44 ± 0.05 | 0.45 ± 0.05 |
| **Choice temperature (β)** | 2.48 ± 0.34 | 3.02 ± 0.37 |
| **Perseverance index (π)** | 0.44 ± 0.06 | 0.36 ± 0.04 |
| **Weighting factor (ω)** | 0.66 ± 0.06 | 0.57 ± 0.04 |

**Supplementary Figure 1. Computational models comparisons.**

(A) Shows the first level choices as a function of the outcome (correct: 1£ in reward condition and 0£ in punishment condition; incorrect: 0£ in reward condition or -1£ in punishment condition) and transition probability of the real and virtual subjects (model simulation) across all conditions and groups: n = 88. Data are shown for the four different computational models. (B) Model-based index and perseveration index (see definition in the text) as a function computational models.

**Supplementary Figure 2.**

The figure depicts the effect of valence (reward versus punishment) in the posterior probability of the model 3 (with perseveration, without model-based) and 4 (with perseveration and model-based) as a function of pharmacological manipulation (BAL = balanced; TD = acute tryptophan depletion). TD causes an increase in the frequency of the model 3 and a concomitant decrease of the frequency of the model 4 in the reward compared to the punishment condition.

**Supplementary references:**

1. Sheehan DV, Lecrubier Y, Sheehan KH, Amorim P, Janavs J, Weiller E *et al.* The Mini-International Neuropsychiatric Interview (M.I.N.I.): the development and validation of a structured diagnostic psychiatric interview for DSM-IV and ICD-10. *J Clin Psychiatry* 1998; **59 Suppl 20:** 22-33;quiz 34-57.

2. Beck AT, Steer RA, Ball R, Ranieri W. Comparison of Beck Depression Inventories -IA and -II in psychiatric outpatients. *Journal of personality assessment* 1996; **67:** 588-597.

3. Spielberger CD. State-Trait anxiety inventory. *Consulting psychologist press, Palo Alto, CA* 1989; **2nd edition**.

4. Bright P, Jaldow E, Kopelman MD. The National Adult Reading Test as a measure of premorbid intelligence: a comparison with stimates derived from demographic variables. *Journal International Neuropsychological Society* 2002; **8:** 847-854.

5. Daunizeau J, Adam V, Rigoux L. VBA: a probabilistic treatment of nonlinear models for neurobiological and behavioural data. *PLoS Computational Biology* 2014**:** 10(11):e1003441.

6. Ardis TC, Cahir M, Elliott JJ, Bell R, Reynolds GP, Cooper SJ. Effect of acute tryptophan depletion on noradrenaline and dopamine in the rat brain. *J Psychopharmacol* 2009 Jan; **23**(1)**:** 51-55.

7. Cox SM, Benkelfat C, Dagher A, Delaney JS, Durand F, Kolivakis T *et al.* Effects of lowerd serotonin transmission on cocaine-induced striatal dopamine response: PET (11C)raclopride study in humans. *British Journal of Psychiatry* 2011; **199**(5)**:** 391-397.

8. Daw N, Kakadeb S, Dayan P. Opponent interactions between serotonin and dopamine. *Neural Networks* 2002; **15**(4-6)**:** 603–616.

9. Cools R, Nakamura K, Daw ND. Serotonin and dopamine: unifying affective, activational, and decision functions. *Neuropsychopharmacology* 2011 Jan; **36**(1)**:** 98-113.

10. Niv Y, Daw ND, Joel D, Dayan P. Tonic dopamine: opportunity coast and the control of response vigor. *Psychopharmacology* 2007; **191**(3)**:** 507-520.

11. Keramati M, Dezfouli A, Piray P. Speed/accuracy trade-off between the habitual and the goal-directed process. *PLoS Computatinal Biology* 2011; **7**(5)**:** e1002055.

12. Dayan P. Instrumental vigor in punishment and reward. *European Journal of Neuroscience* 2012; **35**(7)**:** 1152-1168.
