# Supplementary figures and images for: Valence-dependent influence of serotonin depletion on model-based choice strategy
Source: Mol Psychiatry. 2015 Apr 14;21(5):624–9. doi: 10.1038/mp.2015.46 (PMC4519524; doi:10.1038/mp.2015.46)

(A)

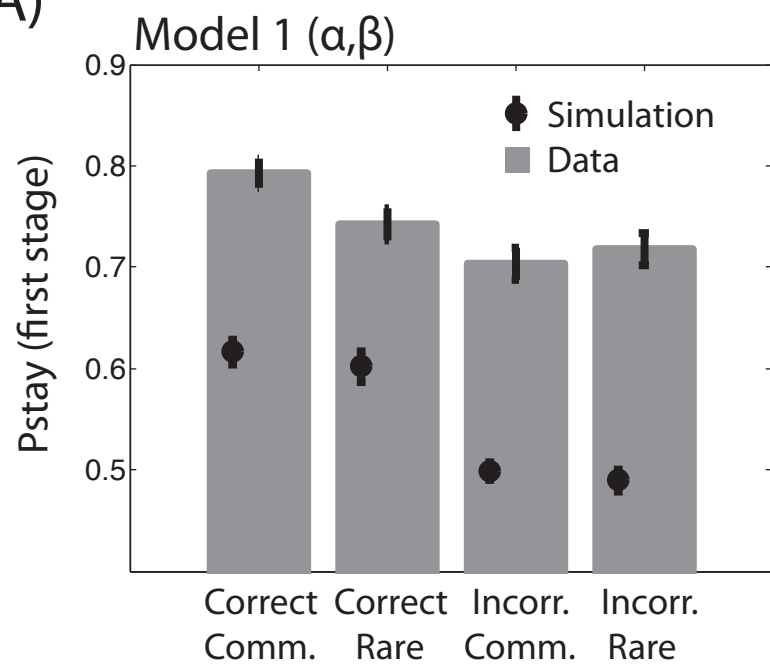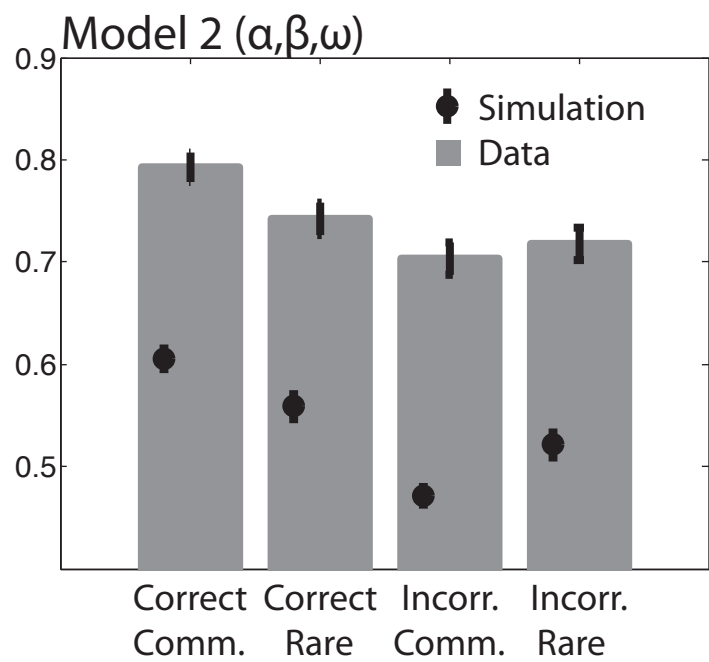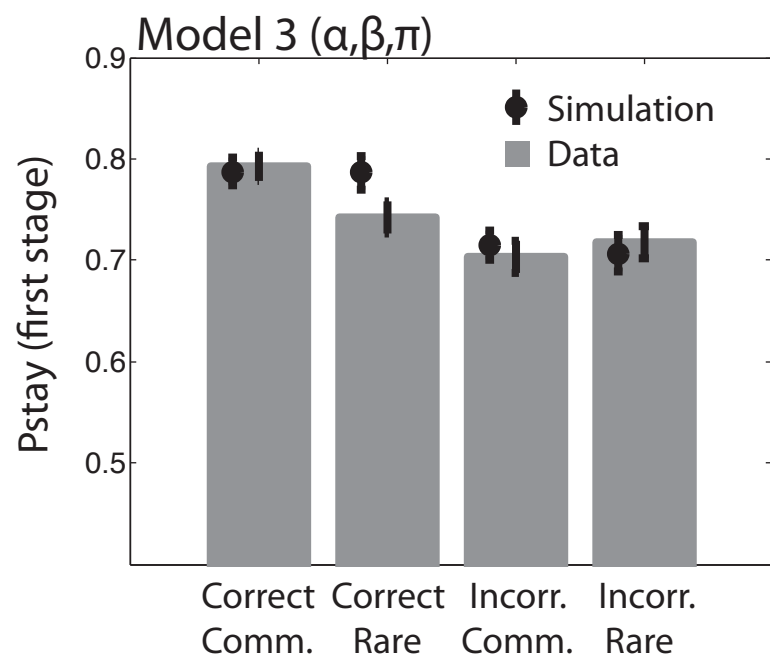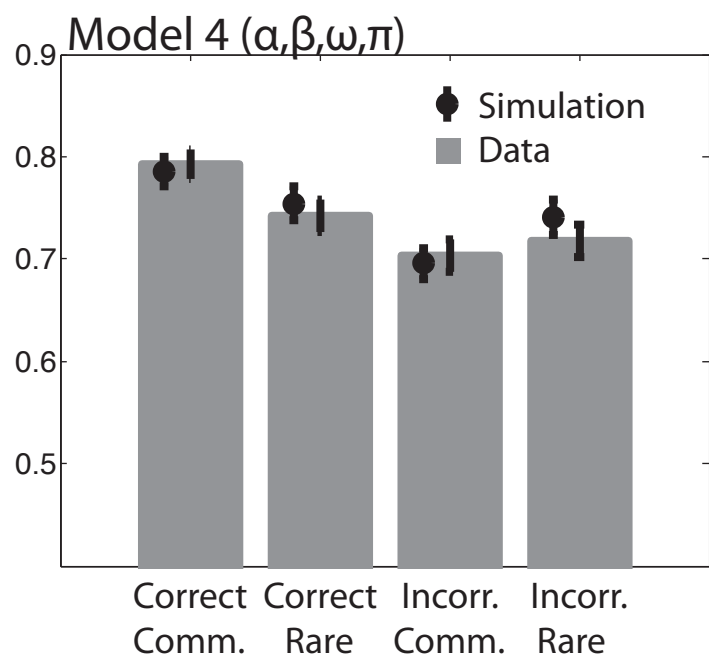

(B)

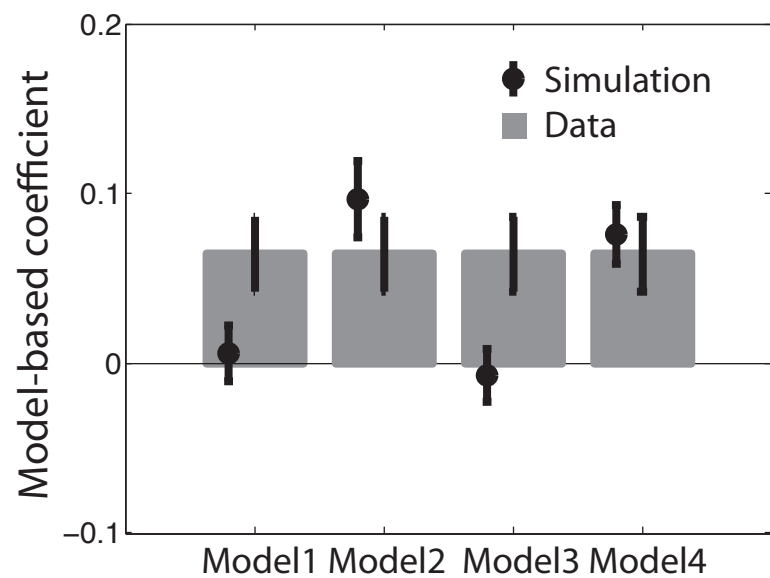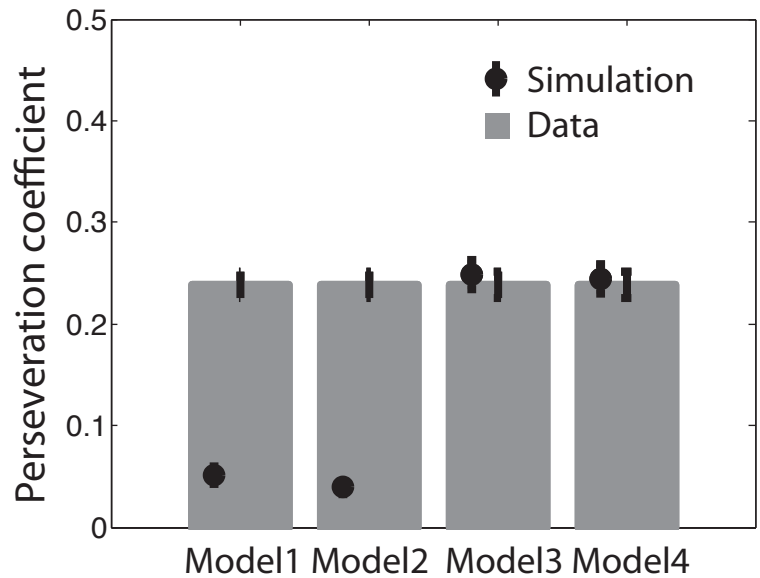

Supplement: Supplementary Figure 1 [file mp201546x2.pdf]

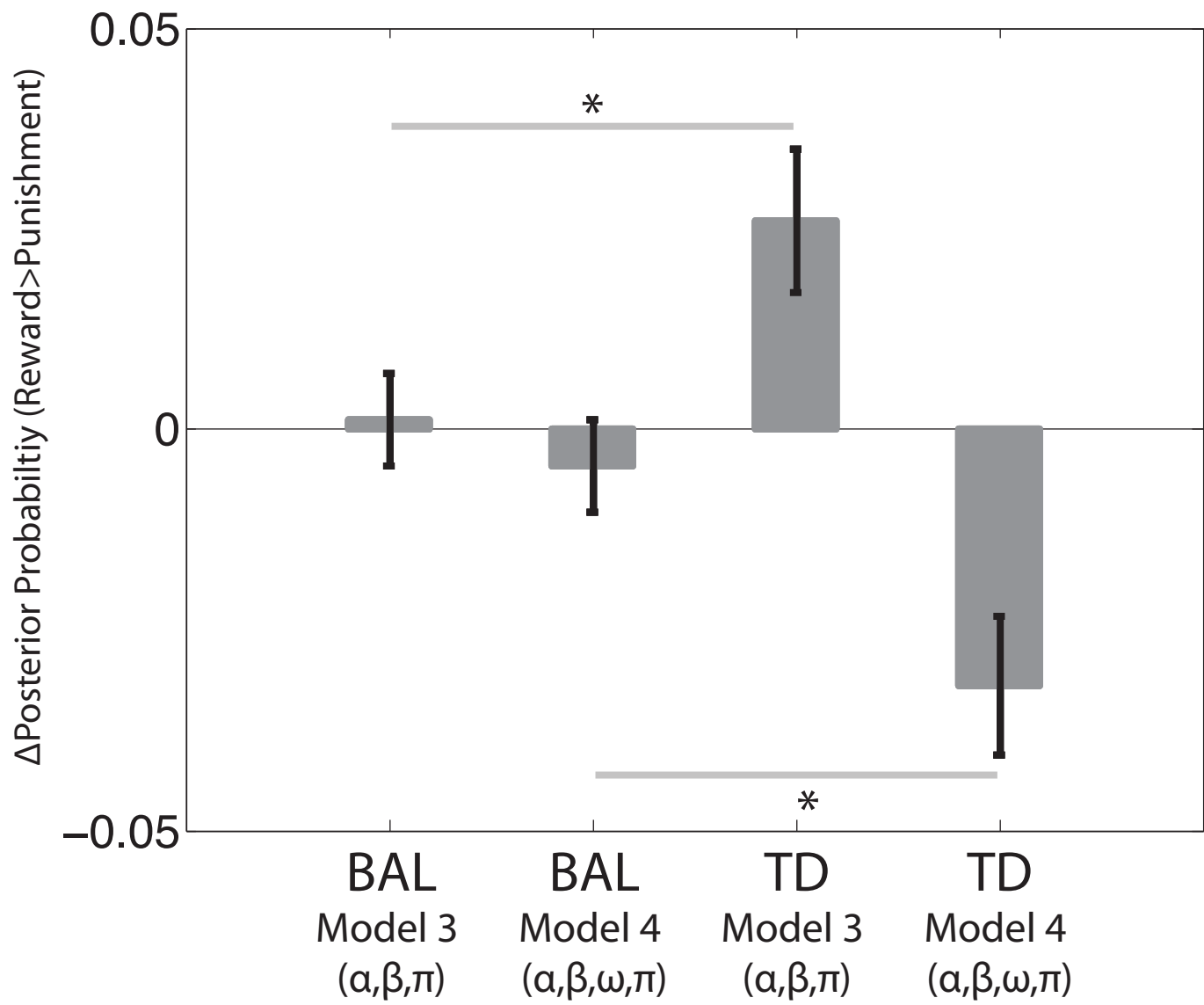

Supplement: Supplementary Figure 2 [file mp201546x3.pdf]
